# The Year's Work of the College of Nursing

**Published:** 1921-05-28

**Authors:** 


### THE MATRONS' AND SISTERS' DEPARTMENT.

### THE YEAR'S WORK OF THE COLLEGE OF NURSING.

The year 1920-21 will always be memorable in the history of the College. The amount of work accomplished, the public recognition and favour accorded to the College, its rapid internal growth, the energy displayed by individual members, in the Centres, in Council and Committee, and by the permanent headquarters staff, all these factors are clearly brought into view in the annual report just issued.

Lord and Lady Cowdray's magnificent gifts of the Club house, at 20 Cavendish Square, and of funds for a College building to be erected on these premises would alone suffice to mark this year as a starting-point in a new stage of development. It is marked also by the fact that the members now exceed the 20,000 total originally forecasted in these columns as a sound test of support from the united profession. The change of constitution, which admits of a subscription from all new members, has taken place at the right moment.

The year's work divides itself conveniently into the consolidation of benefits already assured to members and the launching of new schemes. Among the former we congratulate the members on the continued and remarkable vigour which animates the Local Centres, now numbering twenty-eight.

The quarterly *Bulletin*, well edited and printed, is among the most necessary items of expenditure the funds have to meet, and we are convinced that the cost of despatching it free to all members is well repaid in a hundred directions. The Studentships now open to College members are five in number, and their influence is felt in many directions. The issue last December by the Council of a revised scale of minimum salaries has had a marked effect in introducing better payment in various departments of nursing and, as our columns often testify, has awakened a better conscience in many hitherto indifferent employers of nurses. The Sickness and Illness Insurance Scheme in connection with the Eagle Star and British Dominions Insurance Company has been fairly launched, with a membership of 277. The Loan Fund for helping nurses to obtain further qualifications flourishes as it deserves to do. The Information Bureau, acquiring daily greater importance, is doing valuable work both for members and the public. Legal advice was generously afforded by Sir Charles Russell to forty-one members in professional difficulties.

Of new undertakings the report announces one of the very greatest importance to the profession. This is no less than a scheme for the National Superannuation of Nurses. The Council are tackling this big subject in full consciousness of its magnitude, and we await with interest the first forward steps. Another matter of great importance to the profession is the formation of a Public Health Advisory Committee. Members will read with interest that, as recorded in these columns at the time, the College has been successful in inducing the Central Midwives Board to permit nurses holding the affiliated certificate recognised by the College to sit for the C.M.B. examination on the same terms as other trained nurses after four instead of six months' training. In more directions than we have been able to indicate the influence of the College is penetrating for the moulding of public opinion.

